# Biofilms: Microbial Life on Surfaces

**DOI:** 10.3201/eid0809.020063

**Published:** 2002-09

**Authors:** Rodney M. Donlan

**Affiliations:** * Centers for Disease Control and Prevention, Atlanta, Georgia, USA

**Keywords:** biofilm, bacterial attachment, extracellular polymeric substances, biofilm structure, gene regulation in biofilms, biofilm ecology, biofilms and public health

## Abstract

Microorganisms attach to surfaces and develop biofilms. Biofilm-associated cells can be differentiated from their suspended counterparts by generation of an extracellular polymeric substance (EPS) matrix, reduced growth rates, and the up- and down- regulation of specific genes. Attachment is a complex process regulated by diverse characteristics of the growth medium, substratum, and cell surface. An established biofilm structure comprises microbial cells and EPS, has a defined architecture, and provides an optimal environment for the exchange of genetic material between cells. Cells may also communicate via quorum sensing, which may in turn affect biofilm processes such as detachment. Biofilms have great importance for public health because of their role in certain infectious diseases and importance in a variety of device-related infections. A greater understanding of biofilm processes should lead to novel, effective control strategies for biofilm control and a resulting improvement in patient management.

For most of the history of microbiology, microorganisms have primarily been characterized as planktonic, freely suspended cells and described on the basis of their growth characteristics in nutritionally rich culture media. Rediscovery of a microbiologic phenomenon, first described by van Leeuwenhoek, that microorganisms attach to and grow universally on exposed surfaces led to studies that revealed surface-associated microorganisms (biofilms) exhibited a distinct phenotype with respect to gene transcription and growth rate. These biofilm microorganisms have been shown to elicit specific mechanisms for initial attachment to a surface, development of a community structure and ecosystem, and detachment.

## A Historical Basis

A biofilm is an assemblage of surface-associated microbial cells that is enclosed in an extracellular polymeric substance matrix. Van Leeuwenhoek, using his simple microscopes, first observed microorganisms on tooth surfaces and can be credited with the discovery of microbial biofilms. Heukelekian and Heller (1) observed the “bottle effect” for marine microorganisms, i.e., bacterial growth and activity were substantially enhanced by the incorporation of a surface to which these organisms could attach. Zobell (2) observed that the number of bacteria on surfaces was dramatically higher than in the surrounding medium (in this case, seawater). However, a detailed examination of biofilms would await the electron microscope, which allowed high-resolution photomicroscopy at much higher magnifications than did the light microscope. Jones et al. (3) used scanning and transmission electron microscopy to examine biofilms on trickling filters in a wastewater treatment plant and showed them to be composed of a variety of organisms (based on cell morphology). By using a specific polysaccharide-stain called Ruthenium red and coupling this with osmium tetroxide fixative, these researchers were also able to show that the matrix material surrounding and enclosing cells in these biofilms was polysaccharide. As early as 1973, Characklis (4) studied microbial slimes in industrial water systems and showed that they were not only very tenacious but also highly resistant to disinfectants such as chlorine. Based on observations of dental plaque and sessile communities in mountain streams, Costerton et al. (5) in 1978 put forth a theory of biofilms that explained the mechanisms whereby microorganisms adhere to living and nonliving materials and the benefits accrued by this ecologic niche. Since that time, the studies of biofilms in industrial and ecologic settings and in environments more relevant for public health have basically paralleled each other. Much of the work in the last 2 decades has relied on tools such as scanning electron microscopy (SEM) or standard microbiologic culture techniques for biofilm characterization. Two major thrusts in the last decade have dramatically impacted our understanding of biofilms: the utilization of the confocal laser scanning microscope to characterize biofilm ultrastructure, and an investigation of the genes involved in cell adhesion and biofilm formation.

## Biofilm Defined

**Figure 1:**
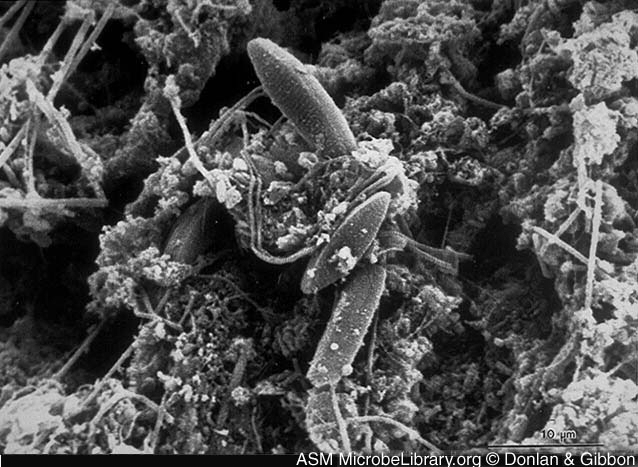
Scanning electron micrograph of a native biofilm that developed on a mild steel surface in an 8-week period in an industrial water system. Rodney Donlan and Donald Gibbon, authors. Licensed for use, American Society for Microbiology MicrobeLibrary. Available from: URL: http://www.microbelibrary.org/

**Figure 2:**
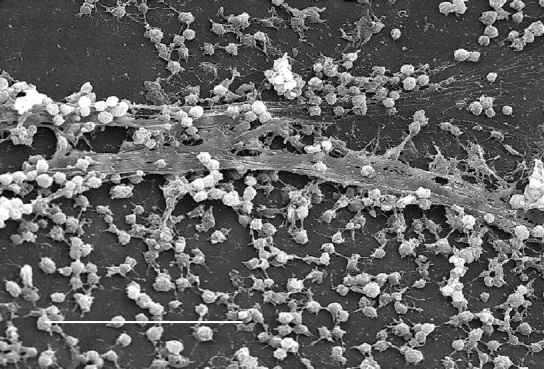
Scanning electron micrograph of a staphylococcal biofilm on the inner surface of an indwelling medical device. Bar, 20 μ. Used with permission of Lippincott Williams & Wilkins.

A biofilm is an assemblage of microbial cells that is irreversibly associated (not removed by gentle rinsing) with a surface and enclosed in a matrix of primarily polysaccharide material. Noncellular materials such as mineral crystals, corrosion particles, clay or silt particles, or blood components, depending on the environment in which the biofilm has developed, may also be found in the biofilm matrix. Biofilm-associated organisms also differ from their planktonic (freely suspended) counterparts with respect to the genes that are transcribed. Biofilms may form on a wide variety of surfaces, including living tissues, indwelling medical devices, industrial or potable water system piping, or natural aquatic systems. The variable nature of biofilms can be illustrated from scanning electron micrographs of biofilms from an industrial water system and a medical device, respectively (Figures 1 and 2). The water system biofilm is highly complex, containing corrosion products, clay material, fresh water diatoms, and filamentous bacteria. The biofilm on the medical device, on the other hand, appears to be composed of a single, coccoid organism and the associated extracellular polymeric substance (EPS) matrix.

## Attachment

The solid-liquid interface between a surface and an aqueous medium (e.g., water, blood) provides an ideal environment for the attachment and growth of microorganisms. A clear picture of attachment cannot be obtained without considering the effects of the substratum, conditioning films forming on the substratum, hydrodynamics of the aqueous medium, characteristics of the medium, and various properties of the cell surface. Each of these factors will be considered in detail.

### Substratum Effects

The solid surface may have several characteristics that are important in the attachment process. Characklis et al. (6) noted that the extent of microbial colonization appears to increase as the surface roughness increases. This is because shear forces are diminished, and surface area is higher on rougher surfaces. The physicochemical properties of the surface may also exert a strong influence on the rate and extent of attachment. Most investigators have found that microorganisms attach more rapidly to hydrophobic, nonpolar surfaces such as Teflon and other plastics than to hydrophilic materials such as glass or metals (7–9). Even though results of these studies have at times been contradictory because no standardized methods exist for determining surface hydrophobicity, some kind of hydrophobic interaction apparently occurs between the cell surface and the substratum that would enable the cell to overcome the repulsive forces active within a certain distance from the substratum surface and irreversibly attach.

### Conditioning Films

A material surface exposed in an aqueous medium will inevitably and almost immediately become conditioned or coated by polymers from that medium, and the resulting chemical modification will affect the rate and extent of microbial attachment. Loeb and Neihof (10) were the first to report the formation of these conditioning films on surfaces exposed in seawater. These researchers found that films were organic in nature, formed within minutes of exposure, and continued to grow for several hours. The nature of conditioning films may be quite different for surfaces exposed in the human host. A prime example may be the proteinaceous conditioning film called “acquired pellicle,” which develops on tooth enamel surfaces in the oral cavity. Pellicle comprises albumin, lysozyme, glycoproteins, phosphoproteins, lipids, and gingival crevice fluid (11); bacteria from the oral cavity colonize pellicle-conditioned surfaces within hours of exposure to these surfaces. Mittelman noted that a number of host-produced conditioning films such as blood, tears, urine, saliva, intervascular fluid, and respiratory secretions influence the attachment of bacteria to biomaterials (12). Ofek and Doyle (13) also noted that the surface energy of the suspending medium may affect hydrodynamic interactions of microbial cells with surfaces by altering the substratum characteristics.

### Hydrodynamics

In theory, the flow velocity immediately adjacent to the substratum/liquid interface is negligible. This zone of negligible flow is termed the hydrodynamic boundary layer. Its thickness is dependent on linear velocity; the higher the velocity, the thinner the boundary layer. The region outside the boundary layer is characterized by substantial mixing or turbulence. For flow regimes characterized as laminar or minimally turbulent, the hydrodynamic boundary layer may substantially affect cell-substratum interactions. Cells behave as particles in a liquid, and the rate of settling and association with a submerged surface will depend largely on the velocity characteristics of the liquid. Under very low linear velocities, the cells must traverse the sizeable hydrodynamic boundary layer, and association with the surface will depend in large part on cell size and cell motility. As the velocity increases, the boundary layer decreases, and cells will be subjected to increasingly greater turbulence and mixing. Higher linear velocities would therefore be expected to equate to more rapid association with the surface, at least until velocities become high enough to exert substantial shear forces on the attaching cells, resulting in detachment of these cells (14) This finding has been confirmed in studies by Rijnaarts et al. (15) and Zheng et al. (16).

### Characteristics of the Aqueous Medium

Other characteristics of the aqueous medium, such as pH, nutrient levels, ionic strength, and temperature, may play a role in the rate of microbial attachment to a substratum. Several studies have shown a seasonal effect on bacterial attachment and biofilm formation in different aqueous systems (17,18). This effect may be due to water temperature or to other unmeasured, seasonally affected parameters. Fletcher (19,20) found that an increase in the concentration of several cations (sodium, calcium, lanthanum, ferric iron) affected the attachment of *Pseudomonas fluorescens* to glass surfaces, presumably by reducing the repulsive forces between the negatively charged bacterial cells and the glass surfaces. Cowan et al. (21) showed in a laboratory study that an increase in nutrient concentration correlated with an increase in the number of attached bacterial cells.

### Properties of the Cell

Cell surface hydrophobicity, presence of fimbriae and flagella, and production of EPS all influence the rate and extent of attachment of microbial cells. The hydrophobicity of the cell surface is important in adhesion because hydrophobic interactions tend to increase with an increasing nonpolar nature of one or both surfaces involved (i.e., the microbial cell surface and the substratum surface). Most bacteria are negatively charged but still contain hydrophobic surface components, as noted by Rosenberg and Kjelleberg (22). Fimbriae, i.e., nonflagellar appendages other than those involved in transfer of viral or bacterial nucleic acids (called pili), contribute to cell surface hydrophobicity. Most fimbriae that have been examined contain a high proportion of hydrophobic amino acid residues (22). Fimbriae play a role in cell surface hydrophobicity and attachment, probably by overcoming the initial electrostatic repulsion barrier that exists between the cell and substratum (23). A number of aquatic bacteria possess fimbriae, which have also been shown to be involved in bacterial attachment to animal cells (23). Rosenburg et al. (24) and Bullitt and Makowski (25) provided evidence for the role of fimbriae in bacterial attachment to surfaces.

Other cell surface properties may also facilitate attachment. Several studies have shown that treatment of adsorbed cells with proteolytic enzymes caused a marked release of attached bacteria (26,27), providing evidence for the role of proteins in attachment. Bendinger et al. (9) found that mycolic acid-containing organisms (*Corynebacterium, Nocardia,* and *Mycobacterium*) were more hydrophobic than were nonmycolic acid-containing bacteria, and increase in mycolic acid chain length generally coincided with increase in hydrophobicity. For most strains tested, adhesion was greater on hydrophobic materials. The O antigen component of lipopolysaccharide (LPS) has also been shown to confer hydrophilic properties to gram-negative bacteria. Williams and Fletcher (28) showed that mutants of *P. fluorescens* lacking the O antigen adhered in greater numbers to hydrophobic materials.

As early as 1971, Marshall et al. (29) provided evidence based on SEM that attached bacteria were associated with the surface via fine extracellular polymeric fibrils. Fletcher et al. (30) found that treatment of attached freshwater bacteria with cations resulted in contraction of the initial adhesives (decrease in the cell distance from the substratum), supporting the idea that this material was an anionic polymer. Cations have been shown to cross-link the anionic groups of polymers (such as polysaccharides), resulting in contraction. Beech and Gaylarde (31) found that lectins inhibited but did not prevent attachment. Glucosidase and N-acetylglucosaminidase reduced attachment for *P.* *fluorescens*, while NAG reduced attachment for *Desulfovibrio desulfuricans*. Lectins preferentially bind to polysaccharides on the cell surface or to the EPS. Binding of lectins by the cells would minimize the attachment sites and affect cell attachment if polysaccharides were involved in attachment. Zottola (32) confirmed the role of polysaccharides in attachment in studies with *Pseudomonas fragi*.

Korber et al. (33) used motile and nonmotile strains of *P. fluorescens* to show that motile cells attach in greater numbers and attach against the flow (backgrowth) more rapidly than do nonmotile strains. Nonmotile strains also do not recolonize or seed vacant areas on a substratum as evenly as motile strains, resulting in slower biofilm formation by the nonmotile organisms. Flagella apparently play an important role in attachment in the early stages of bacterial attachment by overcoming the repulsive forces associated with the substratum.

In light of these findings, cell surface structures such as fimbriae, other proteins, LPS, EPS, and flagella all clearly play an important role in the attachment process. Cell surface polymers with nonpolar sites such as fimbriae, other proteins, and components of certain gram-positive bacteria (mycolic acids) appear to dominate attachment to hydrophobic substrata, while EPS and lipopolysaccharides are more important in attachment to hydrophilic materials. Flagella are important in attachment also, although their role may be to overcome repulsive forces rather than to act as adsorbents or adhesives.

The attachment of microorganisms to surfaces is a very complex process, with many variables affecting the outcome. In general, attachment will occur most readily on surfaces that are rougher, more hydrophobic, and coated by surface “conditioning” films. An increase in flow velocity, water temperature, or nutrient concentration may also equate to increased attachment, if these factors do not exceed critical levels. Properties of the cell surface, specifically the presence of fimbriae, flagella, and surface-associated polysaccharides or proteins, also are important and may possibly provide a competitive advantage for one organism where a mixed community is involved. Table 1 summarizes the variables important in cell attachment and biofilm formation.

**Table 1:** Variables important in cell attachment and biofilm formation

Properties of the substratum	Properties of the bulk fluid	Properties of the cell
		
Texture or roughness	Flow velocity	Cell surface hydrophobicity
Hydrophobicity	pH	Fimbriae
Conditioning film	Temperature	Flagella
	Cations	Extracellular polymeric substances
	Presence of antimicrobial agents	

## Gene Regulation by Attached Cells

Evidence is mounting that up- and down-regulation of a number of genes occurs in the attaching cells upon initial interaction with the substratum. Davies and Geesey (34) demonstrated *algC* up-regulation in individual bacterial cells within minutes of attachment to surfaces in a flow cell system. This phenomenon is not limited to *P. aeruginosa*. Prigent-Combaret et al. (35) found that 22% of these genes were up-regulated in the biofilm state, and 16% were down-regulated. Becker et al. (36) showed that biofilms of *Staphylococcus aureus* were up-regulated for genes encoding enzymes involved in glycolysis or fermentation (phosphoglycerate mutase, triosephosphate isomerase, and alcohol dehydrogenase) and surmised that the up-regulation of these genes could be due to oxygen limitation in the developed biofilm, favoring fermentation. A recent study by Pulcini (37) also showed that *algD*, *algU, rpoS,* and genes controlling polyphosphokinase (PPK) synthesis were up-regulated in biofilm formation of *P. aeruginosa*. Prigent-Combaret et al. (35) opined that the expression of genes in biofilms is evidently modulated by the dynamic physicochemical factors external to the cell and may involve complex regulatory pathways.

## Biofilm Structure

### Extracellular Polymeric Substances

Biofilms are composed primarily of microbial cells and EPS. EPS may account for 50% to 90% of the total organic carbon of biofilms (38) and can be considered the primary matrix material of the biofilm. EPS may vary in chemical and physical properties, but it is primarily composed of polysaccharides. Some of these polysaccharides are neutral or polyanionic, as is the case for the EPS of gram-negative bacteria. The presence of uronic acids (such as D-glucuronic, D-galacturonic, and mannuronic acids) or ketal-linked pryruvates confers the anionic property (39). This property is important because it allows association of divalent cations such as calcium and magnesium, which have been shown to cross-link with the polymer strands and provide greater binding force in a developed biofilm (38). In the case of some gram-positive bacteria, such as the staphylococci, the chemical composition of EPS may be quite different and may be primarily cationic. Hussain et al. (40) found that the slime of coagulase-negative bacteria consists of a teichoic acid mixed with small quantities of proteins.

EPS is also highly hydrated because it can incorporate large amounts of water into its structure by hydrogen bonding. EPS may be hydrophobic, although most types of EPS are both hydrophilic and hydrophobic (39). EPS may also vary in its solubility. Sutherland (39) noted two important properties of EPS that may have a marked effect on the biofilm. First, the composition and structure of the polysaccharides determine their primary conformation. For example, many bacterial EPS possess backbone structures that contain 1,3- or 1,4-β-linked hexose residues and tend to be more rigid, less deformable, and in certain cases poorly soluble or insoluble. Other EPS molecules may be readily soluble in water. Second, the EPS of biofilms is not generally uniform but may vary spatially and temporally. Leriche et al. (41) used the binding specificity of lectins to simple sugars to evaluate bacterial biofilm development by different organisms. These researchers’ results showed that different organisms produce differing amounts of EPS and that the amount of EPS increases with age of the biofilm. EPS may associate with metal ions, divalent cations, other macromolecules (such as proteins, DNA, lipids, and even humic substances) (38). EPS production is known to be affected by nutrient status of the growth medium; excess available carbon and limitation of nitrogen, potassium, or phosphate promote EPS synthesis (39). Slow bacterial growth will also enhance EPS production (39). Because EPS is highly hydrated, it prevents desiccation in some natural biofilms. EPS may also contribute to the antimicrobial resistance properties of biofilms by impeding the mass transport of antibiotics through the biofilm, probably by binding directly to these agents (42).

### Biofilm Architecture

**Figure 3:**
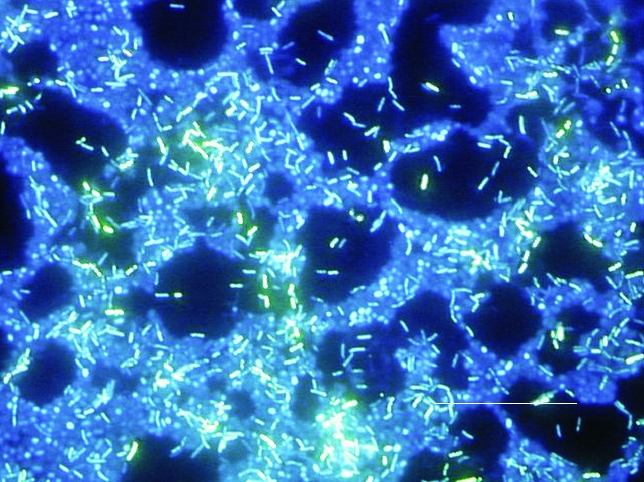
Polymicrobic biofilm grown on a stainless steel surface in a laboratory potable water biofilm reactor for 14 days, then stained with 4,6-diamidino-2-phenylindole (DAPI) and examined by epifluorescence microscopy. Bar, 20 μ.

Tolker-Nielsen and Molin noted that every microbial biofilm community is unique (43) although some structural attributes can generally be considered universal. The term biofilm is in some ways a misnomer, since biofilms are not a continuous monolayer surface deposit. Rather, biofilms are very heterogeneous, containing microcolonies of bacterial cells encased in an EPS matrix and separated from other microcolonies by interstitial voids (water channels) (44). Figure 3 shows a biofilm of *P. aeruginosa, Klebsiella pneumoniae,* and *Flavobacterium* spp. that has developed on a steel surface in a laboratory potable water system. This image clearly depicts the water channels and heterogeneity characteristic of a mature biofilm. Liquid flow occurs in these water channels, allowing diffusion of nutrients, oxygen, and even antimicrobial agents. This concept of heterogeneity is descriptive not only for mixed culture biofilms (such as might be found in environmental biofilms) but also for pure culture biofilms common on medical devices and those associated with infectious diseases. Stoodley et al. (45) defined certain criteria or characteristics that could be considered descriptive of biofilms in general, including a thin base film, ranging from a patchy monolayer of cells to a film several layers thick containing water channels. The organisms composing the biofilm may also have a marked effect on the biofilm structure. For example, James et al. (46) showed that biofilm thickness could be affected by the number of component organisms. Pure cultures of either *K. pneumoniae* or *P*. *aeruginosa* biofilms in a laboratory reactor were thinner (15 μ and 30 μ, respectively), whereas a biofilm containing both species was thicker (40 μ). Jones et al. noted that this could be because one species enhanced the stability of the other.

Biofilm architecture is heterogeneous both in space and time, constantly changing because of external and internal processes. Tolker-Nielsen et al. (47) investigated the role of cell motility in biofilm architecture in flow cells by examining the interactions of *P. aeruginosa* and *P. putida* by confocal laser scanning microscopy. When these two organisms were added to the flow cell system, each organism initially formed small microcolonies. With time, the colonies intermixed, showing the migration of cells from one microcolony to the other. The microcolony structure changed from a compact structure to a looser structure over time, and when this occurred the cells inside the microcolonies were observed to be motile. Motile cells ultimately dispersed from the biofilm, resulting in dissolution of the microcolony.

### Interaction of Particles

Structure may also be influenced by the interaction of particles of nonmicrobial components from the host or environment. For example, erythrocytes and fibrin may accumulate as the biofilm forms. Biofilms on native heart valves provide a clear example of this type of interaction in which bacterial microcolonies of the biofilm develop in a matrix of platelets, fibrin, and EPS (48). The fibrin capsule that develops will protect the organisms in these biofilms from the leukocytes of the host, leading to infective endocarditis. Biofilms on urinary catheters may contain organisms that have the ability to hydrolyze urea in the urine to form free ammonia through the action of urease. The ammonia may then raise the pH at the biofilm-liquid interface, resulting in the precipitation of minerals such as calcium phosphate (hydroxyapatite) and magnesium ammonium phosphate (struvite) (49). These minerals can then become entrapped in the biofilm and cause encrustation of the catheter; cases have been described in which the catheter became completely blocked by this mineral build-up. Minerals such as calcium carbonate, corrosion products such as iron oxides, and soil particles may often collect in biofilms of potable and industrial water systems, providing yet another example of particle interactions with biofilms (50).

## The Established Community: Biofilm Ecology

**Figure 4:**
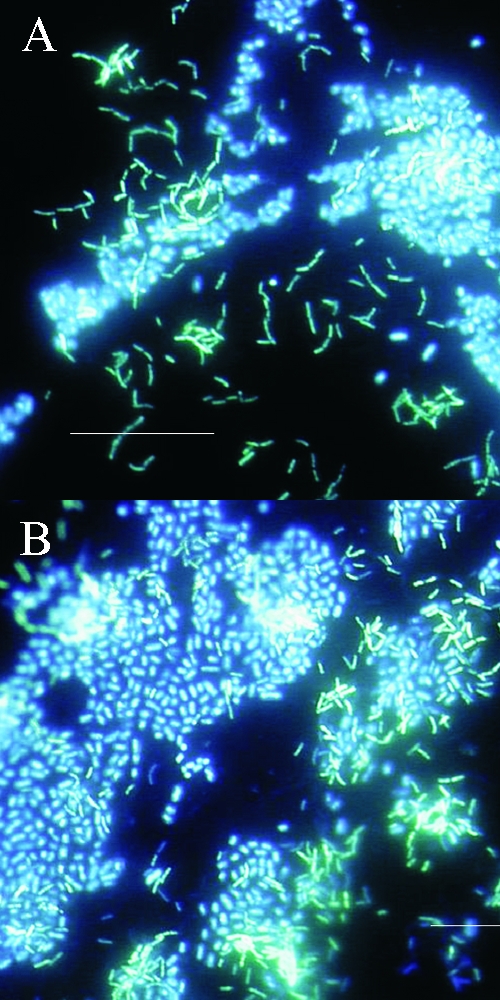
Polymicrobic biofilms grown on stainless steel surfaces in a laboratory potable water biofilm reactor for 7 days, then stained with 4,6-diamidino-2-phenylindole (DAPI) and examined by epifluorescence microscopy. Bar, 20 μ.

The basic structural unit of the biofilm is the microcolony. Proximity of cells within the microcolony (or between microcolonies) (Figure 4A and B) provides an ideal environment for creation of nutrient gradients, exchange of genes, and quorum sensing. Since microcolonies may be composed of multiple species, the cycling of various nutrients (e.g., nitrogen, sulfur, and carbon) through redox reactions can readily occur in aquatic and soil biofilms.

### Gene Transfer

Biofilms also provide an ideal niche for the exchange of extrachromosomal DNA (plasmids). Conjugation (the mechanism of plasmid transfer) occurs at a greater rate between cells in biofilms than between planktonic cells (51–53). Ghigo (54) has suggested that medically relevant strains of bacteria that contain conjugative plasmids more readily develop biofilms. He showed that the F conjugative pilus (encoded by the *tra* operon of the F plasmid) acts as an adhesion factor for both cell-surface and cell-cell interactions, resulting in a three-dimensional biofilm of *Escherichia coli*. Plasmid-carrying strains have also been shown to transfer plasmids to recipient organisms, resulting in biofilm formation; without plasmids these same organisms produce only microcolonies without any further development. The probable reason for enhanced conjugation is that the biofilm environment provides minimal shear and closer cell-to-cell contact. Since plasmids may encode for resistance to multiple antimicrobial agents, biofilm association also provides a mechanism for selecting for, and promoting the spread of, bacterial resistance to antimicrobial agents.

### Quorum Sensing

Cell-to-cell signaling has recently been demonstrated to play a role in cell attachment and detachment from biofilms. Xie et al. (55) showed that certain dental plaque bacteria can modulate expression of the genes encoding fimbrial expression (*fimA*) in *Porphyromonas gingivalis*. *P. gingivalis* would not attach to *Streptococcus cristatis* biofilms grown on glass slides. *P. gingivalis,* on the other hand, readily attached to *S. gordonii*. *S. cristatus* cell-free extract substantially affected expression of *fimA* in *P. gingivalis*, as determined by using a reporter system. *S. cristatus* is able to modulate *P. gingivalis fimA* expression and prevent its attachment to the biofilm.

Davies et al. (56) showed that two different cell-to-cell signaling systems in *P. aeruginosa*, *lasR-lasI* and *rhlR-rhlI,* were involved in biofilm formation. At sufficient population densities, these signals reach concentrations required for activation of genes involved in biofilm differentiation. Mutants unable to produce both signals (double mutant) were able to produce a biofilm, but unlike the wild type, their biofilms were much thinner, cells were more densely packed, and the typical biofilm architecture was lacking. In addition, these mutant biofilms were much more easily removed from surfaces by a surfactant treatment. Addition of homoserine lactone to the medium containing the mutant biofilms resulted in biofilms similar to the wild type with respect to structure and thickness. Stickler et al. (57) also detected acylated homoserine lactone signals homoserine lactone signals in biofilms of gram-negative bacteria on urethral catheters. Yung-Hua et al. (58) showed that induction of genetic competence (enabling the uptake and incorporation of exogenous DNA by transformation) is also mediated by quorum sensing in *S. mutans*. Transformational frequencies were 10–600 times higher in biofilms than planktonic cells.

### Predation and Competition

Bacteria within biofilms may be subject to predation by free-living protozoa, *Bdellovibrio* spp., bacteriophage, and polymorphonuclear leukocytes (PMNs) as a result of localized cell concentration. Murga et al. (59) demonstrated the colonization and subsequent predation of heterotrophic biofilms by *Hartmannella vermiformis*, a free-living protozoon. Predation has also been demonstrated with *Acanthamoeba* spp. in contact lens storage case biofilms (60).

James et al. (46) noted that competition also occurs within biofilms and demonstrated that invasion of a *Hyphomicrobium* sp. biofilm by *P. putida* resulted in dominance by the *P. putida*, even though the biofilm-associated *Hyphomicrobium* numbers remained relatively constant. Stewart et al. (61) investigated biofilms containing *K. pneumonia* and *P. aeruginosa* and found that both species are able to coexist in a stable community even though *P. aeruginosa* growth rates are much slower in the mixed culture biofilm than when grown as a pure culture biofilm. *P. aeruginosa* grow primarily as a base biofilm, whereas *K. pneumoniae* form localized microcolonies (covering only about 10% of the area) that may have greater access to nutrients and oxygen**.** Apparently *P.* *aeruginosa* can compete because it colonizes the surface rapidly and establishes a long-term competitive advantage. *K. pneumoniae* apparently survives because of its ability to attach to the *P. aeruginosa* biofilm, grow more rapidly, and out-compete the *P. aeruginosa* in the surface layers of the biofilm.

### Interactions of Pathogenic Organisms

Several frank bacterial pathogens have been shown to associate with, and in some cases, actually grow in biofilms, including *Legionella pneumophila* (59)*, S. aureus* (62), *Listeria monocytogenes* (63), *Campylobacter* spp*.* (64), *E. coli* O157:H7 (65), *Salmonella typhimurium* (66), *Vibrio cholerae* (67), and *Helicobacter pylori* (68). Although all these organisms have the ability to attach to surfaces and existing biofilms, most if not all appear incapable of extensive growth in the biofilm. This may be because of their fastidious growth requirements or because of their inability to compete with indigenous organisms. The mechanism of interaction and growth apparently varies with the pathogen, and at least for *L. pneumophila,* appears to require the presence of free-living protozoa to grow in the biofilm (59). Survival and growth of pathogenic organisms within biofilms might also be enhanced by the association and metabolic interactions with indigenous organisms. Camper et al. (65) showed that *Salmonella typhimurium* persisted in a model distribution system containing undefined heterotrophic bacteria from an unfiltered reverse osmosis water system for >50 days, which suggests that the normal biofilm flora of this water system provided niche conditions capable of supporting the growth of this organism.

The picture of biofilms increasingly is one in which there is both heterogeneity and a constant flux, as this biological community adapts to changing environmental conditions and the composition of the community.

## Dispersal

Biofilm cells may be dispersed either by shedding of daughter cells from actively growing cells, detachment as a result of nutrient levels or quorum sensing, or shearing of biofilm aggregates (continuous removal of small portions of the biofilm) because of flow effects.

The mechanisms underlying the process of shedding by actively growing cells in a biofilm are not well understood. Gilbert et al. (69) showed that surface hydrophobicity characteristics of newly divided daughter cells spontaneously dispersed from either *E. coli* or *P.* *aeruginosa* biofilms differ substantially from those of either chemostat-intact biofilms or resuspended biofilm cells. These researchers suggested that these differences might explain newly divided daughter cells’ detachment. Hydrophobicity was lowest for the newly dispersed cells and steadily increases upon continued incubation and growth.

Alginate is the major component of the EPS of *P. aeruginosa*. Boyd and Chakrabarty (70) studied alginate lyase production in *P. aeruginosa* to determine whether increased expression of this enzyme affected the size of the alginate molecules (and therefore adhesion of the organisms). Inducing alginate lyase expression substantially decreased the amount of alginate produced, which corresponded with a significant increase in the number of detached cells. The authors suggested that the role of *algL* (the gene cassette for alginate lyase production) in wild type *P. aeruginosa* may be to cause a release of cells from solid surfaces or biofilms, aiding in the dispersal of these organisms. Polysaccharidase enzymes specific for the EPS of different organisms may possibly be produced during different phases of biofilm growth of these organisms.

Detachment caused by physical forces has been studied in greater detail. Brading et al. (71) have emphasized the importance of physical forces in detachment, stating that the three main processes for detachment are erosion or shearing (continuous removal of small portions of the biofilm)**,** sloughing (rapid and massive removal), and abrasion (detachment due to collision of particles from the bulk fluid with the biofilm). Characklis (72) noted that the rate of erosion from the biofilm increases with increase in biofilm thickness and fluid shear at the biofilm-bulk liquid interface. With increase in flow velocity, the hydrodynamic boundary layer decreases, resulting in mixing and turbulence closer to the biofilm surface. Sloughing is more random than erosion and is thought to result from nutrient or oxygen depletion within the biofilm structure (71). Sloughing is more commonly observed with thicker biofilms that have developed in nutrient-rich environments (72). Biofilms in fluidized beds, filters, and particle-laden environments (surface waters) may be subject to abrasion.

Detachment is probably also species specific; *P. fluorescens* disperses and recolonizes a surface (in a flow cell) after approximately 5 h, *V. parahaemolyticus* after 4 h, and *V. harveyi* after only 2 h (73). This process probably provides a mechanism for cells to migrate from heavily colonized areas that have been depleted of surface-adsorbed nutrients to areas more supportive of growth.

The mode of dispersal apparently affects the phenotypic characteristics of the organisms. Eroded or sloughed aggregates from the biofilm are likely to retain certain biofilm characteristics, such as antimicrobial resistance properties, whereas cells that have been shed as a result of growth may revert quickly to the planktonic phenotype.

## A Public Health Perspective

Clinical and public health microbiologists’ recognition that microbial biofilms are ubiquitous in nature has resulted in the study of a number of infectious disease processes from a biofilm perspective. Cystic fibrosis, native valve endocarditis, otitis media, periodontitis, and chronic prostatitis all appear to be caused by biofilm-associated microorganisms. A spectrum of indwelling medical devices or other devices used in the health-care environment have been shown to harbor biofilms, resulting in measurable rates of device-associated infections (74). Table 2 provides a listing of microorganisms commonly associated with biofilms on indwelling medical devices. Biofilms of potable water distribution systems have the potential to harbor enteric pathogens, *L. pneumophila*, nontuberculous mycobacteria, and possibly *Helicobacter pylori*. What is less clear is an understanding of how interaction and growth of pathogenic organisms in a biofilm result in an infectious disease process. Characteristics of biofilms that can be important in infectious disease processes include a) detachment of cells or biofilm aggregates may result in bloodstream or urinary tract infections or in the production of emboli, b) cells may exchange resistance plasmids within biofilms, c) cells in biofilms have dramatically reduced susceptibility to antimicrobial agents, d) biofilm-associated gram-negative bacteria may produce endotoxins, and e) biofilms are resistant to host immune system clearance. Please refer to the online appendix for an expanded discussion of each of these mechanisms (URL: http://www.cdc.gov/ncid/eid/vol8/no9donlan.htm).

**Table 2:** Microorganisms commonly associated with biofilms on indwelling medical devices

Microorganism	Has been isolated from biofilms on
*Candida albicans*	Artifical voice prosthesis Central venous catheter Intrauterine device
Coagulase-negative staphylococci	Artificial hip prosthesis Artificial voice prosthesis Central venous catheter Intrauterine device Prosthetic heart valve Urinary catheter
*Enterococcus* spp.	Artificial hip prosthesis Central venous catheter Intrauterine device Prosthetic heart valve Urinary catheter
*Klebsiella pneumoniae*	Central venous catheter Urinary catheter
*Pseudomonas aeruginosa*	Artificial hip prosthesis Central venous catheter Urinary catheter
*Staphylococcus aureus*	Artificial hip prosthesis Central venous catheter Intrauterine device Prosthetic heart valve

## A Prospectus for Future Research

Research on microbial biofilms is proceeding on many fronts, with particular emphasis on elucidation of the genes specifically expressed by biofilm-associated organisms, evaluation of various control strategies (including medical devices treated with antimicrobial agents and antimicrobial locks) for either preventing or remediating biofilm colonization of medical devices, and development of new methods for assessing the efficacy of these treatments. Research should also focus on the role of biofilms in antimicrobial resistance, biofilms as a reservoir for pathogenic organisms, and the role of biofilms in chronic diseases. The field of microbiology has come to accept the universality of the biofilm phenotype. Researchers in the fields of clinical, food and water, and environmental microbiology have begun to investigate microbiologic processes from a biofilm perspective. As the pharmaceutical and health-care industries embrace this approach, novel strategies for biofilm prevention and control will undoubtedly emerge. The key to success may hinge upon a more complete understanding of what makes the biofilm phenotype so different from the planktonic phenotype.
